# Biopsy with same-session MRI-guided laser interstitial thermal therapy versus biopsy alone in patients with primary unresectable glioblastoma: a multicentre randomised controlled trial

**DOI:** 10.1016/j.lanepe.2026.101696

**Published:** 2026-05-07

**Authors:** Céline L.G. Neutel, Christiaan G. Overduin, Pieter van Eijsden, Anne Rijpma, Gerjon Hannink, Janneke P.C. Grutters, Hilko Ardon, Rutger Balvers, Koos Hovinga, Michiel Wagemakers, Philip de Witt Hamer, Pierre A. Robe, Maroeska M. Rovers, Mark ter Laan

**Affiliations:** a Department of Neurosurgery, Radboud University Medical Centre, Nijmegen, the Netherlands; b Department of Medical Imaging, Radboud University Medical Centre, Nijmegen, the Netherlands; c Department of Neurosurgery, University Medical Centre Utrecht, Utrecht, the Netherlands; d Science Department IQ Health, Radboud University Medical Centre, Nijmegen, the Netherlands; e Department of Neurosurgery, Elisabeth TweeSteden Ziekenhuis Tilburg, Tilburg, the Netherlands; f Department of Neurosurgery, Brain Tumour Centre, Erasmus University Medical Center, the Netherlands; g Department of Neurosurgery, MUMC+, Maastricht, the Netherlands; h Department of Neurosurgery, University Medical Centre Groningen, the Netherlands; i Department of Neurosurgery, Amsterdam University Medical Centre, Amsterdam, the Netherlands

**Keywords:** Glioblastoma, LITT, Laser interstitial thermal therapy, Stereotactic laser ablation, Randomised controlled trial

## Abstract

**Background:**

Glioblastoma (GBM) is an aggressive malignancy with poor outcomes, particularly when resection is infeasible. Laser Interstitial Thermal Therapy (LITT) offers a minimally invasive cytoreductive option, though robust evidence remains limited. The aim of the EMITT trial (NCT05318612) was to evaluate LITT in these patients. However, the study was terminated early following funding retraction due to slow patient inclusion. Despite being underpowered, its findings provide valuable insights into LITT’s potential role.

**Methods:**

In this non-blinded multicentre randomised controlled trial, adult patients with unresectable GBM were randomised 1:1 to biopsy or biopsy with same-session LITT followed by standard of care. Patients were recruited by seven Dutch neurosurgical hospitals. Primary outcomes were overall survival and health-related quality of life (HR-QoL) at 5 months post-randomization. Minimum follow-up was 18 months or until death.

**Findings:**

Patients were included between April 19, 2022, and March 19, 2024. Twenty-nine patients were included; three were excluded post-randomization, leaving twenty-six for intention-to-treat analyses: fourteen in the control group and twelve in the intervention group. Median survival was 4.8 months (95%CI 3.4–not estimable) in controls and 7.8 months (95%CI 2.58–not estimable) in intervention. Mean QLQ-C30 summary scores at 5 months were 77 (SD 11) and 81 (SD 11).

**Interpretation:**

Definitive conclusions regarding (cost-)effectiveness are precluded by small sample size and baseline imbalances. This work highlights the need for larger prospective studies to establish LITT’s effectiveness in this population and lessons learned may inform the design of future trials.

**Funding:**

Funding by the Dutch Healthcare Institute and 10.13039/501100001826ZonMw.

**Research in context**

**Evidence before this study**

Before the initiation of this study, we systematically searched PubMed and Embase for studies using search terms related to “laser interstitial thermal therapy”, “glioblastoma”, “newly diagnosed”, and synonyms thereof (search date 17 December 2020). Of the 835 identified publications, 11 studies met the inclusion criteria for this systematic review. The majority were retrospective case series or uncontrolled cohort studies, were judged to have a serious or critical risk of bias according to ROBINS-I and provided very low-quality evidence as assessed by GRADE. Reported median overall survival ranged from 4.1 to 32 months, progression-free survival from 2 to 31 months, and mean complication rates were ∼34%. Peri- and postoperative reporting, confounders, and patient-reported outcomes were inconsistent, and no studies were randomised; despite seemingly favourable outcomes, the evidence remains of low quality. Since the systematic review, eight additional studies have been published examining LITT in high-grade glioma (search date 12 April 2026), including a prospective controlled pilot study by our own group. The findings from these studies indicate that the evidence base for LITT in unresectable GBM has remained largely unchanged, and high-quality prospective data are still lacking.

Our randomised pilot study in patients with newly diagnosed, unresectable GBM, compared biopsy alone with biopsy plus same-session MR-guided LITT followed by standard chemoradiotherapy assessing feasibility of a randomised controlled trial in this population. In 15 patients, follow-up adherence was high, adjuvant therapy was not delayed, and the procedure demonstrated acceptable safety.

**Added value of this study**

The randomised controlled EMITT trial was designed to provide high-level evidence on the effectiveness of LITT in newly diagnosed, unresectable GBM. Due to slow accrual and withdrawal of funding, the trial was prematurely terminated, leaving 26 patients for analysis. In these patients the median survival appeared longer in the intervention group, and HR-QoL was comparable, but definitive conclusions are precluded by the small sample size and baseline imbalances. Nevertheless, this study provides the first prospective randomised evidence in this patient population.

**Implications of all the available evidence**

Owing to the limited sample size, no definitive conclusions regarding the (cost-)effectiveness of LITT can be drawn from our results. Consequently, the role of LITT in this patient population remains uncertain, and further large-scale, adequately powered prospective studies are required to establish its (cost-)effectiveness. Since the initiation of this trial, LITT in primary GBM has continued to attract international interest, despite the persistent lack of strong evidence. The results and experiences from the EMITT trial are valuable for future trial design.

## Introduction

Glioblastoma (GBM) is the most aggressive primary brain tumour in adults, characterized by rapid growth, invasive nature, and poor prognosis. Incidence of GBM ranges between 3.19 and 4.17 cases per 100.000 person-years.^1^, ^2^, ^3^ According to the Dutch Cancer Registry (NKR/IKNL), approximately 870 new cases of glioblastoma are diagnosed annually in the Netherlands.^4^ Despite advancements in neurosurgical techniques, radiotherapy, and chemotherapy in the past decades, the median survival for patients with GBM remains dismal. Patients undergoing surgical resection followed by adjuvant therapy generally have the most favourable prognosis. Reported median survival ranges from 13.5 to 18.8 months, contingent on the extent of resection,^5^ while nationwide data from the Netherlands indicate a median survival of 12.9 months.^6^ However, surgical resection is not feasible in approximately 30% of patients, primarily due to unfavourable tumour location with subsequent risk of surgical deterioration of patients’ condition.^7^ These patients can only undergo biopsy followed by adjuvant treatment, and consequently potentially miss the survival benefit associated with cytoreduction, resulting in a worse prognosis. Reported median survival is approximately 9.2 months in the literature, and ranges from 4.6 to 5.6 months in the Netherlands.^5^^,^^6^^,^^8^^,^^9^ This is further supported by data from the Dutch Quality Registry Neurosurgery, which report a median survival of 12.6 months for patients undergoing surgical resection, compared to approximately 5.6 months for those receiving only biopsy.^9^ This underscores the need for alternative treatments to improve outcomes, particularly for those in whom standard surgical approaches are not feasible.

Laser Interstitial Thermal Therapy (LITT) is a minimally invasive technique used to treat various types of intracranial lesions and is increasingly being applied worldwide. This procedure is performed under Magnetic Resonance (MR) thermometry guidance, which allows for real-time thermal control and targeted treatment. The advantages of MR-guided LITT include its minimally invasive nature, the ability to precisely target (difficult-to-reach) intracranial lesions, and thus reduced damage to surrounding healthy brain tissue.^10^ Furthermore, LITT can be repeated if necessary, making it a versatile treatment option for recurrent tumours.^11^

For patients with unresectable GBM, LITT represents a new treatment option.^12^, ^13^, ^14^ The technique offers an alternative for achieving cytoreduction in otherwise unresectable cases, potentially improving survival and quality of life. A systematic review by our group suggests that LITT in combination with standard adjuvant treatment may be associated with a median survival ranging from 3.3 to 32.3 months in patients with unresectable GBMs, which would be an improvement compared to only biopsy.^15^ Moreover, the minimally invasive nature of LITT translates to reduced operative risks, possibly making treatment available at higher risk locations.^11^

Despite its potential, the evidence supporting LITT’s (cost-)effectiveness remains limited.^15^^,^^16^ We therefore initiated a multi-centre randomised controlled trial to compare addition of LITT to current standard of care in patients with primary unresectable GBM. Despite the setup of a national multicentre RCT, patient recruitment proved challenging, causing significant enrolment delays. Consequently, the funding body terminated support early, resulting in a much smaller sample size than planned and precluding definitive conclusions. Despite this limitation, we believe our results offer valuable insights into the potential of LITT for treating primary unresectable GBM.

## Methods

This trial was conducted according to the study design outlined in the study protocol published in August 2023^17^ and reported according to the CONSORT guideline.^18^ The CONSORT checklist is provided as Supplementary item 1.

### Study design and participants

The study was a non-blinded multicentre randomised controlled trial. Seven Dutch neurosurgical hospitals participated in this trial. All patients assigned to the intervention group were referred to one of the two centres that performed LITT (Radboud university medical centre or UMC Utrecht) to receive the intervention. For both groups, adjuvant treatment (chemotherapy, radiotherapy, a combination thereof, or no additional therapy) was administered according to the advice of the local tumour board based on (inter)national guidelines. Ethical approval for this study was granted by the Medical Ethical Committee of the Arnhem-Nijmegen region, the Netherlands, on 21 March 2022 (NL79202.091.21). All methods were carried out in accordance with relevant guidelines and regulations. All amendments were approved by the Medical Ethical Committee region Arnhem-Nijmegen. Patient representatives actively contributed to the development of the study protocol and met annually with the research team to receive progress updates and offer feedback. The trial was registered on ClinicalTrials.gov (NCT05318612) on 1 April 2022.

Adult patients with a radiologically suspected primary GBM were eligible for participation in this trial. If the local tumour board or neurosurgeon recommended a biopsy only due to the tumour’s unfavourable location, or patients’ condition, or if the patient declined conventional tumour resection but remained open to less invasive approaches such as LITT, the patient was presented to the study expert panel. All patients included in this study had unresectable tumours as determined by local MDT and confirmed by expert panel assessment, while no patients with resectable tumours were included. This panel, consisting of three neurosurgeons, two of whom perform LITT, evaluated the patient’s suitability. Patients were eligible for inclusion if deemed suitable by at least two of three experts. The inclusion criteria required participants to be at least 18 years of age, have a radiological diagnosis of a supratentorial primary GBM deemed ineligible for surgical resection, have the potential for laser ablation of at least 70% of the tumour, and have a Karnofsky Performance Score (KPS) of 70 or higher. The planned ablation threshold of ≥70% was chosen by extrapolating from surgical literature on extent of resection, with the dual aim of avoiding non-therapeutic small ablations and maximizing the eligible study population. Exclusion criteria were pregnancy, contraindications for MRI or anaesthesia, and an inability to complete Dutch-language questionnaires. Detailed inclusion and exclusion criteria are available in the published study protocol.^17^ If deemed suitable, the patient was then approached for informed consent.

### Randomisation and masking

After obtaining informed consent, patients were included and randomised, using randomised permuted blocks^4^^,^^6^ through the web-based module CastorEDC,^19^ by the principal investigator of the including centre. Participants were assigned in a 1:1 ratio to either the biopsy only (control) group or to the biopsy combined with LITT (intervention) group, stratified by centre.

### Procedures

Patients in the control group received standard care, which was a biopsy followed by adjuvant treatment. Those in the intervention group underwent a biopsy with subsequent same-session MR-guided LITT followed by adjuvant treatment.

Patients in the control group were typically admitted the day before the procedure and underwent a neuronavigation MRI or CT scan. On the day of the procedure, a stereotactic biopsy was performed. Afterwards, patients remained in the neurosurgery department and were usually discharged the following day. All neurosurgeons at each participating centre were allowed to perform this procedure, according to their standard operating procedures.

Patients in the intervention group were admitted the day before the procedure, and a neuronavigation MRI or CT scan was performed. An ablation plan was developed based on this MRI or CT, and one to three trajectories were designed to optimize coverage. If it was determined at that time that the tumour had grown significantly compared to its size at the moment of inclusion, making a 70% ablation unachievable, the LITT procedure was not carried out, and the patient only received a biopsy. All LITT procedures were carried out using the Visualase Thermal Therapy System (Medtronic, USA). In brief, a stereotactic biopsy was performed in accordance with standard clinical protocol. A frozen section was submitted for pathological evaluation. Upon confirmation of glial tissue, the probes were inserted. Subsequently, the patient was transferred to the MRI suite, where the ablation procedure was conducted. A detailed description of the LITT procedure can be found in the published study protocol.^17^ Following the procedure, patients were admitted to the neurosurgery department and were typically discharged on the first or second postoperative day. Three neurosurgeons in the two LITT centres were trained to perform this procedure in the context of this trial.

### Outcomes

This study hypothesized that adding LITT would improve survival without significantly affecting health-related quality of life. The co-primary outcomes were health-related quality of life (HR-QoL) and overall survival (OS). HR-QoL was measured at 5 months post-randomization, using the EORTC QLQ-C30 and QLQ-BN20 scores which would be assessed for non-inferiority. OS would be assessed for superiority. The 5-month time point was selected for the HR-QoL analysis because it corresponded to the anticipated median survival in the control group, ensuring that at least half of the control group remained evaluable. Moreover, by 5 months post-randomization, short-term effects of adjuvant treatment on quality of life were expected to have subsided, while longer-term effects could still be adequately captured.

Secondary outcomes were healthcare costs, generic and health-related quality of life throughout the study period (EQ5D-5L, EORTC QLQ-C30 and QLQ-BN20), progression free survival (PFS), disease specific survival (DSS), effects on adjuvant treatment, ablation percentage and complication rates.

Clinical and imaging follow-up adhered to standard care, with no additional scans conducted as part of the study. Patients had follow-up visits every 3 months, MRIs at 3 and 6 months, and later if clinically relevant. In cases where no adjuvant treatment was initiated or it was discontinued, follow-up visits and follow-up scans might not have been performed which is in line with standard care. Decisions regarding adjuvant treatment initiation and continuation and follow-up MRIs were made in consultation between the patient and their treating physician.

Data from follow-up visits were obtained from the notes of treating physicians. No additional visits or contacts were made beyond standard care. This approach aimed to minimize additional burden on patients while aligning closely with actual practice.

EORTC QLQ-C30, BN20, and EQ5D-5L questionnaires were completed at inclusion, within 72 h after the biopsy (+LITT) procedure, and one month after randomization. During follow-up, these questionnaires were administered monthly for the first six months, and then at 12 and 18 months, and annually up to a maximum of 64 months.

Questionnaires were filled out online via a secure web-based module (Castor EDC), if needed with help from a relative, friend or caregiver. The coordinating study researcher managed follow-up and questionnaire administration. Patients and their families were encouraged to complete the questionnaires, though incomplete responses were anticipated due to the patients’ clinical conditions and disease progression. Incomplete responses were accounted for in the power calculation.

Patients were followed up until one of the following occurred: (i) death, (ii) end of study or (iii) the patient’s decision to withdraw from the study.

Data collection and management were conducted using Castor EDC. Data was sourced from electronic patient files and digital questionnaires. Local PIs and if applicable other local researchers received training in data collection procedures. Depending on the number of included patients, monitoring visits were conducted at each study site up to twice a year.

### Statistical analysis

The co-primary outcomes were HR-QoL (QLQ-C30 + BN20) at five months and overall survival, which informed the sample size calculation. For overall survival, a hazard ratio of 0.5 was assumed, corresponding to an increase in median survival from 5.1 to 10.2 months, requiring 92 patients (46 per arm) to be included. For HR-QoL, a non-inferiority margin of 10 points was used, requiring 192 patients (96 per arm) to detect non-inferiority with 80% power at 0.05 significance level. Accounting for a 5% post-randomization exclusion rate, a 5% pre-intervention dropout rate, and a 10% loss to follow-up, the final required sample size was calculated to be 238 patients. A more detailed explanation and calculation for the sample size is provided in the published study protocol.^17^

All analyses and results are descriptive and exploratory in nature. No formal hypothesis testing was performed due to the limited sample size resulting from premature study termination. The primary analyses were conducted using the intention-to-treat (ITT) approach. Additionally, survival outcomes were examined using the as-treated (AT) and per-protocol (PP) approaches. For survival analysis, median survival times with corresponding 95% confidence intervals (CIs) were calculated, and Kaplan–Meier curves were generated for both groups. Quality of life (QoL) at the 5-month time point was assessed using mean scores and standard deviations (SDs) for all dimensions of the QLQ-C30 and QLQ-BN20 questionnaires.

Adverse events (AEs) were reported as counts within each AE category for both study groups. To assess QoL over time, estimated means of EQ-5D-5L scores derived from mixed models were presented, along with their corresponding confidence intervals. Ablation volumes, defined as the overlap between the ablated volume and the pre-operative tumour volume, were reported as median values with ranges for the intervention group, along with the percentage of ablation, defined as the percentage of overlap between the ablated volume and pre-operative tumour volume, with ranges for each group. Ablation volumes and percentages of ablation were measured using BrainLab Software. In accordance with recommendations from a recent systematic review for defining ablation volumes after LITT,^20^ the pre-operative tumour volume was delineated on pre-LITT contrast-enhanced T1-weighted (ceT1) MRI, and the ablated volume was delineated on the immediate post-LITT ceT1 MRI. The enhancing rim surrounding the central hypoperfused perfusion deficit was included in the ablation measurements. Ablation volumes are reported in cubic centimetres (cc), and the extent of ablation (EOA) is expressed as a percentage. Volume measurements were done by one researcher and checked by the neurosurgeons who executed the procedures. The effects of adjuvant treatment were described as counts of patients who initiated and completed treatment, type of adjuvant treatment, and the time intervals between the procedure and the start of adjuvant treatment were reported as medians with interquartile ranges (IQRs). Progression-free survival and disease-specific survival times were reported as medians with 95% confidence intervals (CIs) for each group. All analyses were conducted using R (version 4.5.0, R Foundation for Statistical Computing, Vienna, Austria). Analyses were conducted using available data only, without imputation of missing values.

### Role of the funding source

The funder was involved in the study design but had no role in the conduct of the trial, data analysis, or reporting of the results. The sponsor reserved the right to discontinue the study at any time.

## Results

**Fig. 1:**
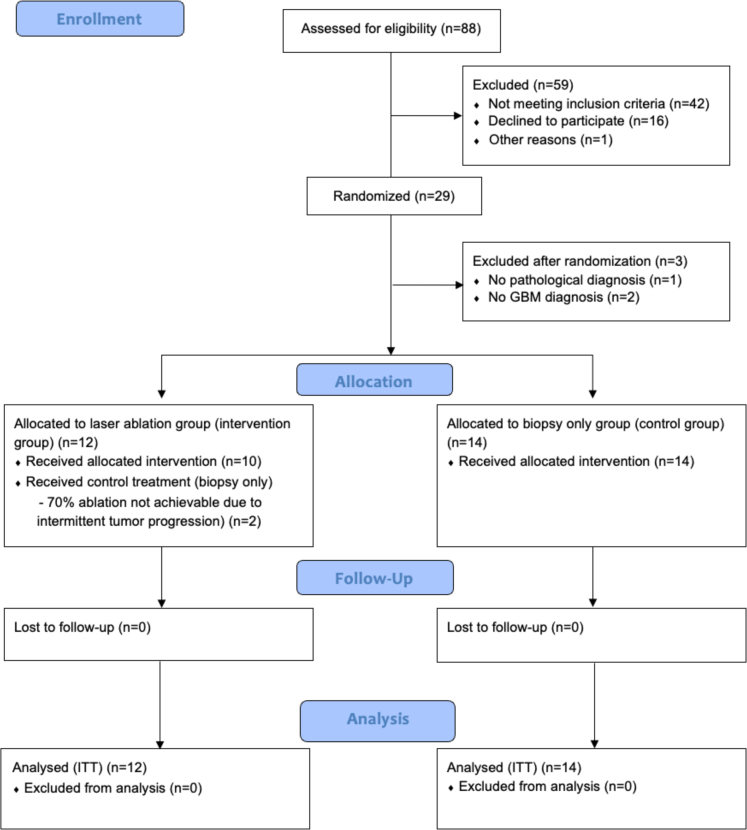
CONSORT flow diagram.

Patients were enrolled in the trial from April 19, 2022, until March 19, 2024. Due to the low inclusion rate, the sponsor withdrew funding, leading to the termination of patient inclusion in March 2024. At that point, a total of 29 patients had been enrolled. Of all these patients inclusion was approved unanimously by the expert panel; there was no disagreement among its members regarding their eligibility for inclusion. Of these, 15 patients were randomised to the control group and 14 to the intervention group. Following inclusion, three patients were subsequently excluded: one from the control group and two from the intervention group. In two cases, a diagnosis other than GBM was identified based on either frozen section analysis or final pathological assessment. One patient experienced significant clinical progression, precluding the possibility of obtaining a biopsy and thereby preventing definitive pathological confirmation of GBM. As a result, data from 26 patients were available for all analyses: 14 in the control group and 12 in the intervention group. Due to tumour progression between time of randomization and the LITT procedure, rendering the minimum required ablation of 70% unachievable, two patients randomised to the intervention group did not undergo LITT and instead received the control treatment (cross-over). One patient in the control group underwent surgical tumour resection 36 days after biopsy. This procedure was performed because the tumour had exhibited significant growth following the biopsy, extending toward the cortical surface and resulting in significant mass effect. This changed the risk/benefit ratio of the resection, making the tumour amenable to surgical resection. This patient is included in the control group in the intention-to-treat and as-treated analyses and excluded from the per-protocol analysis. Of the 26 patients included, 25 were diagnosed with IDH wild type glioblastoma, while one patient in the control group was diagnosed with IDH-mutant astrocytoma, grade 4. Last moment of follow-up was on August 6, 2025. At that time, two patients were still alive. An overview of patient enrolment, allocation, follow-up, and analysis are shown in the CONSORT flow chart (Fig. 1). Table 1 shows the baseline characteristics of the two study groups.

**Table 1:** Baseline characteristics of both study groups.

	Biopsy-only	Laser ablation
N	14	12
Age (median [IQR])	69 [60, 71]	66.5 [60.6, 73.5]
Self-reported gender (women) (%)	3 (21.4)	3 (25.0)
Karnofsky performance score (KPS) (%)		
70	3 (21.4)	2 (16.7)
80	4 (28.6)	3 (25.0)
90	6 (42.9)	6 (50.0)
100	1 (7.1)	1 (8.3)
Dexamethasone use (%)		
No	8 (57.1)	7 (58.3)
Yes	5 (35.7)	4 (33.3)
Missing	1 (7.1)	1 (8.3)
Anti-epileptic drug use (%)		
No	13 (92.9)	8 (66.7)
Yes	1 (7.1)	3 (25.0)
Missing	0 (0.0)	1 (8.3)
Neurological symptoms (%)		
No	1 (7.1)	0 (0.0)
Yes	13 (92.9)	12 (100)
Location of tumour (%)		
Basal ganglia	6 (42.9)	1 (8.3)
Frontal	1 (7.1)	3 (25.0)
Temporal	2 (14.3)	2 (16.7)
Parietal	2 (14.3)	1 (8.3)
Occipital	0 (0.0)	1 (8.3)
Insula	0 (0.0)	1 (8.3)
Thalamus	2 (14.3)	2 (16.7)
Corpus callosum	3 (21.4)	1 (8.3)
Multifocality (%)		
Focal	11 (78.6)	10 (83.3)
Multifocal	3 (21.4)	2 (16.7)
Tumour volume on contrast-enhanced T1 MRI (cm^3^) (median [IQR])	13.90 [6.52, 20.02]	4.84 [2.77, 10.43]
EQ5D score (median (Q1, Q3))	0.82 (0.64, 0.88)	0.87 (0.82, 0.88)
QLQ-C30 summary score (median (Q1, Q3))	90 (85, 94)	82 (71, 90)

**Fig. 2:**
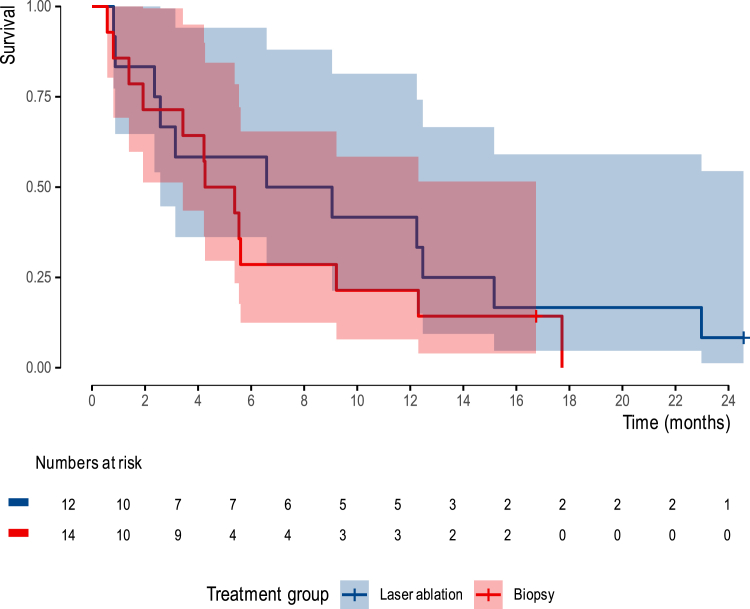
Kaplan–Meier curve for survival (ITT), measured from randomization, in the intervention (red) and control (blue) groups.

In the control group, a median survival of 4.8 months (95%CI 3.4 – not estimable (NE)) was observed, while the intervention group showed a median survival of 7.8 months (95%CI 2.6 – NE). The number of events was insufficient to estimate the upper confidence limit. The Kaplan–Meier curves (Fig. 2) provide a visual representation of this survival data. As-treated and per-protocol analyses are provided as Supplementary item 2.

A total of 14 patients (7 in the control group and 7 in the intervention group) were alive at 5 months post-randomization. Six of the seven patients in the control group and all patients in the intervention group completed the 5-month questionnaire (completion rate 13/14, 93%).

Table 2 shows QoL results for both study groups. For the EORTC QLQ-C30 global health status (QL), the control group showed a mean score 67 (SD = 20) and the intervention group had a mean score of 61 (SD = 28). In terms of the EORTC QLQ-C30 summary score, the control group had a mean score of 77 (SD = 11), and the intervention group had a mean score of 81 (SD = 11).

**Table 2:** Quality of life 5 months after randomization.

Characteristic	Biopsy-only (N = 6)	Laser ablation (N = 7)
QLQ-C30		
Global health status^a^	67 (20)	61 (28)
Functional scales^a^		
Physical	53 (41)	76 (29)
Role	36 (43)	76 (30)
Emotional	68 (14)	77 (26)
Cognitive	75 (17)	71 (21)
Social	56 (33)	57 (33)
Symptom scales^b^		
Fatigue	44 (25)	46 (34)
Nasea and vomiting	2.8 (6.8)	2.4 (6.3)
Pain	0 (0)	7 (13)
Dyspnoea	6 (14)	5 (13)
Insomnia	28 (25)	10 (25)
Appetite loss	0 (0)	14 (18)
Constipation	6 (14)	14 (26)
Diarrhoea	0 (0)	4.8 (12.6)
Financial difficulties	0 (0)	10 (25)
QLQ-C30 summary score	77 (11)	81 (11)
QLQ-BN20		
Symptom scale^b^		
Future uncertainty	36 (23)	33 (27)
Visual disorder	19 (23)	11 (13)
Motor dysfunction	43 (38)	13 (16)
Communication deficit	37 (40)	22 (31)
Headaches	0 (0)	10 (16)
Seizures	6 (14)	14 (26)
Drowsiness	22 (17)	29 (30)
Itchy skin	17 (28)	10 (16)
Hair loss	11 (17)	14 (18)
Weakness of legs	17 (41)	14 (18)
Bladder control	28 (25)	19 (26)

All scores are presented as estimated means with SDs.

a In the function scales and summary score higher scores represent better health.

b In the symptom scales higher scores represent worse health.

A total of 103 adverse events (AEs), including 34 serious adverse events (SAEs), were reported during the study. Most AEs were related to disease progression, such as an increase in cognitive dysfunction and a decline in mobility. All adverse events and serious adverse events per study group are depicted in Table 3.

**Table 3:** Adverse events and serious adverse events per study group.

	Biopsy	Laser ablation
All adverse events	56	47
(Worsening of) symptoms related to GBM	42	35
Symptoms not related to GBM	14	12
Serious adverse events	20	14
Death due to disease progression	12	9
Death due to other cause	1	2
Hospitalization	5	1
Clinical deterioration leading to KPS < 70	2	2
Other	0	0

Of the 34 SAEs reported, 24 concerned patient deaths. Except for one death attributed to COVID-19, one classified as resulting from acute cardiac decompensation, and one due to progressive dyspnoea, all other fatalities were classified as related to disease progression. Classification of the cause of death was performed by the responsible physician, in most cases the general practitioner.

No interim safety analyses were performed by the DSMB, as the number of (serious) adverse events (possibly) related to the surgical procedure, remained below the threshold prespecified in the DSMB charter required for such analyses.

**Fig. 3:**
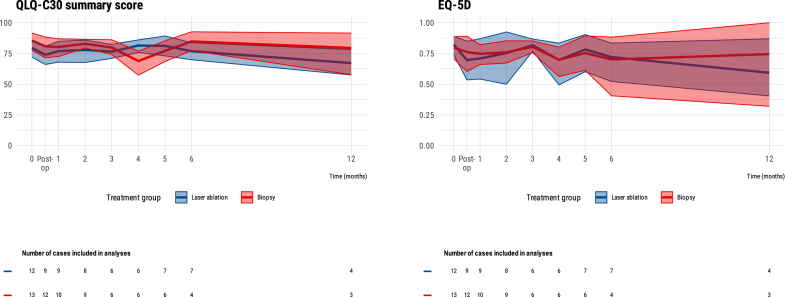
QoL scores with bootstrapped 95% CIs over time across study groups.

Fig. 3 displays the progression of QoL throughout the study period for both study groups. No differences between the groups were observed over time.

The median tumour volume of LITT patients on the day prior to the procedure was 8.8 cm^3^. The median ablation volume including the contrast-enhancing rim was 8.85 cm^3^. The median ablated volume, being the intersection between the ablation volume and pre-operative tumour volume, was 6.25 cm^3^ (range 0.54–29.7 cm^3^), resulting in a median extent of ablation of 79% (range 10–98%). In two patients the threshold of 70% ablation was not reached. In one of them, only 10% of the tumour could be ablated, most likely due to a central necrotic component and proximity to the ventricle, both of which may have acted as a heat sink, thereby absorbing thermal energy and limiting effective heat distribution, despite multiple ablation attempts.

A total of 14 out of 26 patients started adjuvant treatment: 7 out of 14 patients in the control group and 7 out of 12 patients in the intervention group. Among the five intervention group patients who did not receive adjuvant treatment, one patient was a cross-over and did thus not undergo LITT. Three patients in the control group did not complete their adjuvant treatment, with the reasons being toxicity for one patient and disease progression for two patients. In the intervention one out of 7 patients did not complete the adjuvant treatment, with the reason being toxicity. Table 4 shows the different types of adjuvant treatments per group, along with the number of completed adjuvant treatments.

**Table 4:** Adjuvant treatment plans and completion of adjuvant treatment per group.

	Biopsy-only	Laser ablation
N	14	12
Adjuvant treatment (%)		
Stupp protocol (30 × 2 Gy radiation with concomitant temozolomide followed by 6 cycles of adjuvant temozolomide)	3 (21.4)	4 (33.3)
Perry protocol (15 × 2, 67 Gy radiation with concomitant temozolomide followed by 6 cycles of adjuvant temozolomide)	2 (14.3)	2 (16.7)
Chemotherapy	2 (14.3)	0 (0.0)
Radiotherapy	0 (0.0)	1 (8.3)
Other clinical trial	0 (0.0)	0 (0.0)
None	7 (50.0)	5 (41.7)
Adjuvant treatment completed (%)	4 (57.1)	6 (85.7)

In the control group, the median interval between the biopsy and the initiation of adjuvant treatment was 34 days. In the intervention group, the median interval between LITT and the commencement of adjuvant treatment was 41 days.

Two patients in the control group and two patients in the intervention group initiated adjuvant treatment later than the recommended 6-week timeframe, respectively at 44 and 63 days, and at 45 and 55 days, following biopsy or LITT.

**Fig. 4:**
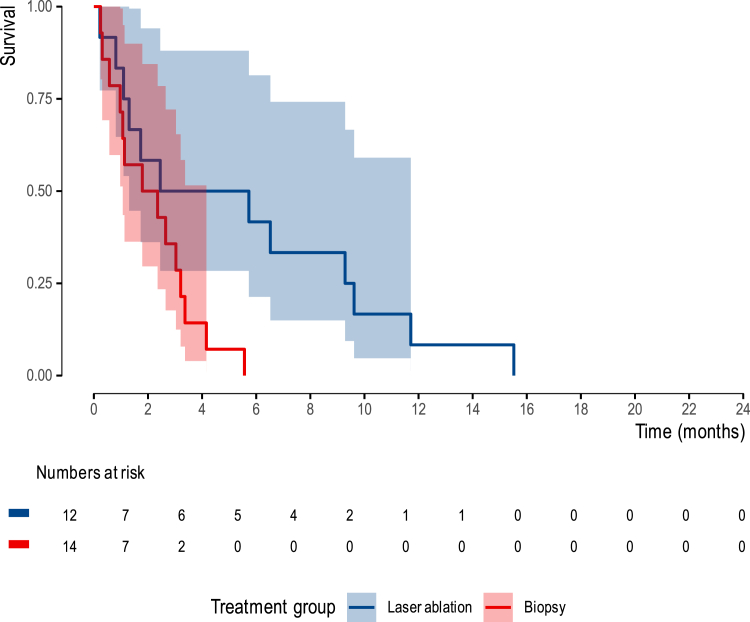
Kaplan–Meier curve for progression-free survival, measured from randomization, in the intervention and control groups.

In the control group, the median progression-free survival was 2 months (95%CI 1–4.2), whereas the intervention group demonstrated a median progression-free survival of 4 months (95%CI 1.3 – NE). The Kaplan–Meier curves (Fig. 4) visually depict the progression-free survival outcomes.

Median DSS in the control group was 4.8 months (95%CI 3.4 – NE), compared to 9 months (95%CI 2.6 – NE) in the intervention group. A total of four patients of which three in the control group and one in the intervention group ultimately chose for euthanasia. One patient in the control group and two patients in the intervention group died from causes other than disease progression. In the control group one patient died due to a COVID-19 infection. In the intervention group one patient died due to cardiac decompensation, and one patient died due to progressive dyspnoea.

Individual patient-level data on baseline characteristics, length of hospital stay, and outcomes are provided in Supplementary item 3.

## Discussion

This study is the first RCT that aimed to evaluate the effectiveness of LITT in patients with primary GBM. Despite early termination, which resulted in a limited sample size of 26 patients for analysis, the findings provide valuable insights into the potential effectiveness of LITT. The median OS was 7.8 months (95%CI 2.6 – NE) in the intervention group compared to 4.8 months (95%CI 3.4 – NE) in the control group. Additionally, five months after randomization, the mean QLQ-C30 summary score was 81 (SD = 11) in the intervention group and 77 (SD = 11) in the control group. Within this limited study population, no conclusions can be drawn regarding the effectiveness of LITT.

The observed median overall survival and HR-QoL in the intervention group appear to be in line with previous reports on patients undergoing LITT for primary high-grade glioma. Several earlier studies, predominantly retrospective case series without a control group, have reported median survival ranging between approximately 6 to 10 months following LITT, depending on patient selection and tumour characteristics, with one more recent systematic review reporting a median survival ranging from 3.3 to 32.3 months.^15^^,^^16^^,^^21^ In these reports, HR-QoL outcomes seemed preserved post-procedure, although formal QoL assessments were often lacking. Our findings are thus consistent with these studies, although with the added strength of a controlled design and more profound measurement of QoL. Notably, differences in survival across studies may reflect heterogeneity in baseline characteristics, such as tumour location and size. Furthermore, the adverse events observed in our cohort, being primarily neurological deterioration or transient worsening of symptoms, are also comparable to those previously described. Earlier reports have suggested that most complications following LITT are attributable to (transient) perilesional oedema rather than thermal damage itself, a finding that is supported by our results.^22^^,^^23^ Furthermore, analysis of our cohort indicated that the majority of observed adverse events were related to disease progression.

This study has several important strengths. Foremost, the intention to conduct a randomised controlled trial provides a methodological strength, as it aims to minimize bias and enables a more rigorous evaluation of the intervention compared to previously published studies. In addition, the inclusion of HR-QoL as a primary outcome provides valuable insight into the broader impact of LITT on both patient well-being and clinical outcomes. Another notable strength is that the novel procedure was performed exclusively in two of the seven participating centres, thereby ensuring procedural consistency and reducing inter-operator variability during the early phase of implementation. Structured proctoring and regular case discussions further supported procedural standardization and mutual learning. This collaborative approach took place not only between the two participating LITT centres but also involved guidance from international centres with more extensive experience in this technique. Finally, all outcomes were assessed using predefined, standardized protocols, enhancing the internal validity and reproducibility of the study findings.

Some limitations should also be acknowledged. First, due to the early termination of our trial, the sample size was too small to draw definitive conclusions regarding the effectiveness of LITT in this patient population. Due to the small final study population and the consequently limited added value of comparative statistical tests, not all analyses including cost-effectiveness analyses, were conducted as originally planned in the study protocol. Despite conservative calculations of the potential sample size and conducting a feasibility pilot study before the initiation of this trial, inclusion lagged leading to the prematurely termination of the trial. We found the most important reasons for this being the clinical condition of GBM patients, the tumour being too large to be able to ablate at least 70% and patients declined participation in the trial. In addition, three patients were excluded after randomization. In accordance with the study protocol, these patients were not included in the intention-to-treat population. At the time of trial design, we anticipated that such rare occurrences would be evenly distributed between groups in a fully accrued study. However, given the ultimately limited sample size, the proportional impact of these exclusions may be greater than originally anticipated.

Second, one of the inclusion criteria stipulated that at least 70% of the tumour must be amenable to ablation which was successfully achieved in 8 out of 10 cases on post-operative evaluation. Although this threshold is also supported by more recent evidence,^13^ literature also suggests that the clinical benefit of LITT is most expected when complete tumour ablation is achieved, reinforcing the importance of striving for complete ablation.^13^^,^^24^ However, to optimize study feasibility, a balance must be found between the ideal scenario (i.e., 100% tumour ablation) and practical considerations, as only a small subset of GBMs is sufficiently small to allow complete ablation which would lead to a too limited potential study population. Importantly, broadening this inclusion criterium may lead to an underestimation of the potential benefit of LITT.

Third, one patient in the control group was ultimately diagnosed with an IDH-mutant astrocytoma. Although the study protocol permitted inclusion of all WHO grade 4 tumours, in retrospect it would have been methodologically preferable to restrict eligibility to patients with IDH-wildtype glioblastoma to ensure a more homogeneous study population.

Finally, it is important to acknowledge the disparity in median baseline tumour volume, tumour location and adjuvant treatment between groups. Given that the tumour volume in the control group was markedly larger than in the intervention group (median 13.9 cm^3^ versus 4.84 cm^3^), tumours were more frequently located in the basal ganglia in the control group and more patients started and completed adjuvant treatment in the intervention group, questions arise as to whether the observed effect can be attributed solely to the intervention. These imbalances may bias the treatment effect. With a larger number of inclusions, baseline imbalances could have been accounted for; however, the current limited sample size precludes such adjustments.

As stated before, patient recruitment proved to be the principal challenge of this trial, despite a deliberately conservative estimate of the eligible population and the completion of a prior feasibility pilot study. We believe that multiple factors contributed to the lower-than-anticipated inclusion rate. Most importantly, patients encountered in routine clinical practice often presented in a poorer clinical condition than suggested by preliminary screening, rendering many ultimately ineligible for participation. In addition, a larger proportion of patients than expected, both compared with projections and with the pilot study, declined trial participation or even active treatment altogether. Within the Dutch healthcare context, this may partly reflect the preference among both clinicians and patients to prioritize quality of life over life prolongation. Certain aspects of the trial design likely contributed to the challenges in recruitment. Unlike the single-centre pilot study, the EMITT trial employed a multicentre design, which may have introduced variability in the interpretation of inclusion and exclusion criteria, heterogeneity in assessing GBM resectability, and opportunities for clinician-driven patient selection, ultimately reducing the number of patients deemed eligible. Especially because multicentre or even multinational collaboration is essential given the limited population coordination of patient recruitment and monitoring of adherence to screening procedures is essential. Finally, the requirement that at least 70% of the tumour volume be amenable to ablation excluded a subset of patients with larger lesions. Nevertheless, we consider this criterion justified since decreasing the targeted ablation percentage would likely reduce clinical benefit and is not justified by the current literature.

The findings of this prematurely terminated randomised trial indicate that LITT might be feasible and safe in a carefully selected subset of patients with primary glioblastoma who are not candidates for surgical resection within a controlled study setting. Given the limited sample size our results cannot establish LITT as an effective treatment for the underlying disease. While this study shows potential benefits in terms of survival and preservation of quality of life in this study, it remains uncertain whether these observed advantages reflect true effects rather than chance, and, if confirmed, whether they would be substantial enough to justify broader implementation, particularly in this vulnerable patient group. Future research, including the pooling of data across studies, may help to strengthen the evidence base and allow for more robust evaluation of these effects. Furthermore, it is unclear whether this potential benefit outweighs the extra costs of LITT. A randomised controlled trial in this population appears feasible; however, persistently slow and complex recruitment highlights the need for multicentre or multinational collaboration, clear communication on eligibility criteria, monitoring of screening processes, and realistic timelines to ensure adequate accrual. As a temporary alternative, large-scale international prospective studies or registries may provide valuable data.

In conclusion, our findings show that within this restricted sample size, the median survival of patients with unresectable GBM receiving the current standard of care, being biopsy followed by standard of care, was 4.8 months, compared to 7.8 months for those receiving LITT plus standard of care, with mean QLQ-C30 summary scores at 5 months after randomization of 77 in the control group, and 81 in the intervention group. As a result of withdrawal of funding, and consequently early termination of patient inclusion and the study itself, the study remains underpowered for firm conclusions. Large RCTs or large-scale, multicentre prospective cohort studies conducted across multiple countries may provide the sample size required to achieve sufficient statistical power to further evaluate the effectiveness of LITT in primary inoperable GBM patients.

## Contributors

CN: Conceptualization, methodology, project management and administration, data collection, data verification together with study monitor, access to raw data, data analysis, writing (original draft), decision to submit for publication.

CO: Conceptualization, methodology, data collection, access to raw data, writing (review and editing), supervision, funding acquisition, decision to submit for publication.

PvE: Conceptualization, methodology, writing (review and editing), funding acquisition.

AR: Conceptualization, methodology, access to raw data, writing (review and editing).

GH: Conceptualization, methodology, data verification, access to raw data, data analysis, writing (review and editing).

JG: Conceptualization, methodology, writing (review and editing), funding acquisition.

HA: Data collection, writing (review and editing).

RB: Data collection, writing (review and editing).

KH: Data collection, writing (review and editing).

MW: Data collection, writing (review and editing).

PWH: Data collection, writing (review and editing).

PR: Conceptualization, methodology, writing (review and editing), funding acquisition.

MR: Conceptualization, methodology, access to raw data, writing (review and editing), supervision, funding acquisition, decision to submit for publication.

MtL: Conceptualization, methodology, access to raw data, writing (review and editing), supervision, funding acquisition, decision to submit for publication.

All authors read and approved the final manuscript.

## Data sharing statement

The datasets generated and/or analysed during the current study will be made available with restricted access in the Radboud Data Repository upon publication. Requests for access will be checked by the principal investigator and/or the data against the conditions for sharing the data as described in the signed Informed Consent. The repository will include the full dataset, associated metadata, and a comprehensive data dictionary to facilitate reuse and interpretation.

## Declaration of interests

MR, MtL and CO received funding from the Dutch Healthcare Institute (“Veelbelovende Zorg”)/ZonMW for conduction of this trial. MtL, CO, PvE and CN received partial financial support from Medtronic to attend a Visualase training. MtL is a board member of the Dutch Society of Neuro Oncology (LWNO). HA is a board member of the Dutch Society for Neurosurgery (chair of the Committee on Quality). MR received an honorarium for giving a lecture at Siemens Healthineers. PR was member of an advisory panel for Strycker.

All other authors declare that they have no conflicts of interests.

## Appendix A Supplementary data

Supplementary items 1–3
