# Supplementary material for: Biopsy with same-session MRI-guided laser interstitial thermal therapy versus biopsy alone in patients with primary unresectable glioblastoma: a multicentre randomised controlled trial
Source: Lancet Reg Health Eur. 2026 May 7;66:101696. doi: 10.1016/j.lanepe.2026.101696 (PMC13156677; doi:10.1016/j.lanepe.2026.101696)
Supplement: Supplementary items 1–3 [file mmc1.pdf]

Supplementary item 1: CONSORT checklist

Section/topic	No	CONSORT 2025 checklist item description	Reported on page no.
**Title and abstract**			
Title and structured abstract	1a	Identification as a randomised trial	1
	1b	Structured summary of the trial design, methods, results, and conclusions	2 - 3
**Open science**			
Trial registration	2	Name of trial registry, identifying number (with URL) and date of registration	1
Protocol and statistical analysis plan	3	Where the trial protocol and statistical analysis plan can be accessed	5
Data sharing	4	Where and how the individual de-identified participant data (including data dictionary), statistical code and any other materials can be accessed	27
Funding and conflicts of interest	5a	Sources of funding and other support (eg, supply of drugs), and role of funders in the design, conduct, analysis and reporting of the trial	1, 28
	5b	Financial and other conflicts of interest of the manuscript authors	28
**Introduction**			
Background and rationale	6	Scientific background and rationale	4 - 5
Objectives	7	Specific objectives related to benefits and harms	4 - 5
**Methods**			

Patient and public involvement	8	Details of patient or public involvement in the design, conduct and reporting of the trial	27
Trial design	9	Description of trial design including type of trial (eg, parallel group, crossover), allocation ratio, and framework (eg, superiority, equivalence, non-inferiority, exploratory)	5 - 11 + protocol
Changes to trial protocol	10	Important changes to the trial after it commenced including any outcomes or analyses that were not prespecified, with reason	10 - 11
Trial setting	11	Settings (eg, community, hospital) and locations (eg, countries, sites) where the trial was conducted	6 + protocol
Eligibility criteria	12a	Eligibility criteria for participants	6 + protocol
	12b	If applicable, eligibility criteria for sites and for individuals delivering the interventions (eg, surgeons, physiotherapists)	7 + protocol
Intervention and comparator	13	Intervention and comparator with sufficient details to allow replication. If relevant, where additional materials describing the intervention and comparator (eg, intervention manual) can be accessed	7 + protocol
Outcomes	14	Prespecified primary and secondary outcomes, including the specific measurement variable (eg, systolic blood pressure), analysis metric (eg, change from baseline, final value, time to event), method of aggregation (eg, median, proportion), and time point for each outcome	8 + protocol
Harms	15	How harms were defined and assessed (eg, systematically, non-systematically)	8 - 9, 11 + protocol
Sample size	16a	How sample size was determined, including all assumptions supporting the sample size calculation	9 + protocol
	16b	Explanation of any interim analyses and stopping guidelines	11 + protocol
Randomisation:			5 - 6
Sequence generation	17a	Who generated the random allocation sequence and the method used	

	17b	Type of randomisation and details of any restriction (eg, stratification, blocking and block size)	5 - 6
			**Reported on page no.**
Allocation concealment mechanism	18	Mechanism used to implement the random allocation sequence (eg, central computer/telephone; sequentially numbered, opaque, sealed containers), describing any steps to conceal the sequence until interventions were assigned	5 - 6
Implementation	19	Whether the personnel who enrolled and those who assigned participants to the interventions had access to the random allocation sequence	5 - 6
Blinding	20a	Who was blinded after assignment to interventions (eg, participants, care providers, outcome assessors, data analysts)	5
	20b	If blinded, how blinding was achieved and description of the similarity of interventions	N.A.
Statistical methods	21a	Statistical methods used to compare groups for primary and secondary outcomes, including harms	10 - 11
	21b	Definition of who is included in each analysis (eg, all randomised participants), and in which group	12
	21c	How missing data were handled in the analysis	10 - 11
	21d	Methods for any additional analyses (eg, subgroup and sensitivity analyses), distinguishing prespecified from post hoc	N.A.
**Results**			
Participant flow, including flow diagram	22a	For each group, the numbers of participants who were randomly assigned, received intended intervention, and were analysed for the primary outcome	12 - 13
	22b	For each group, losses and exclusions after randomisation, together with reasons	12 - 13
Recruitment	23a	Dates defining the periods of recruitment and follow-up for outcomes of benefits and harms	12
	23b	If relevant, why the trial ended or was stopped	5, 12

Intervention and comparator delivery	24a	Intervention and comparator as they were actually administered (eg, where appropriate, who delivered the intervention/comparator, how participants adhered, whether they were delivered as intended (fidelity))	12
	24b	Concomitant care received during the trial for each group	20
Baseline data	25	A table showing baseline demographic and clinical characteristics for each group	14
Numbers analysed, outcomes and estimation	26	For each primary and secondary outcome, by group:<br>● the number of participants included in the analysis<br>● the number of participants with available data at the outcome time point<br>● result for each group, and the estimated effect size and its precision (such as 95% confidence interval)<br>● for binary outcomes, presentation of both absolute and relative effect size	12 - 22
Harms	27	All harms or unintended events in each group	18 -19
Ancillary analyses	28	Any other analyses performed, including subgroup and sensitivity analyses, distinguishing pre-specified from post hoc	N.A.
**Discussion**			
Interpretation	29	Interpretation consistent with results, balancing benefits and harms, and considering other relevant evidence	23 - 26
Limitations	30	Trial limitations, addressing sources of potential bias, imprecision, generalisability, and, if relevant, multiplicity of analyses	23 - 26

Citation: Hopewell S, Chan AW, Collins GS, Hróbjartsson A, Moher D, Schulz KF, et al. CONSORT 2025 Statement: updated guideline for reporting randomised trials. BMJ. 2025; 388:e081123. https://dx.doi.org/10.1136/bmj-2024-081123
© 2025 Hopewell et al. This is an Open Access article distributed under the terms of the Creative Commons Attribution License (https://creativecommons.org/licenses/by/4.0/), which permits unrestricted use, distribution, and reproduction in any medium, provided the original work is properly cited.

*We strongly recommend reading this statement in conjunction with the CONSORT 2025 Explanation and Elaboration and/or the CONSORT 2025 Expanded Checklist for important clarifications on all the items. We also recommend reading relevant CONSORT extensions. See www.consort-spirit.org.

Supplementary item 2: As-treated and per-protocol survival analyses

As-treated survival analysis

	N	Deaths	Median	0.95 LCL	0.95 UCL
Laser ablation	10	9	9.4	2.6	NE
Biopsy only	16	15	4.8	1.9	12.3

NE = not estimable

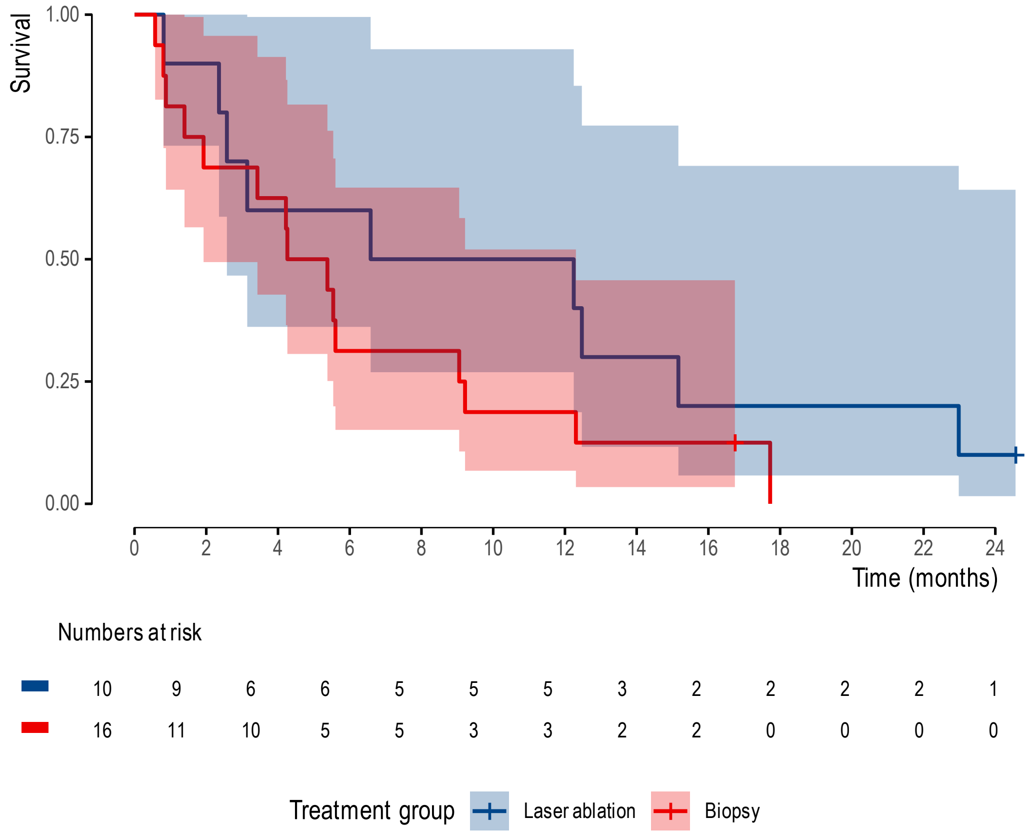

Per-protocol survival analysis

	N	Deaths	Median	0.95 LCL	0.95 UCL
Laser ablation	10	9	9.4	2.6	NE
Biopsy only	14	13	4.3	1.9	NE

NE = not estimable

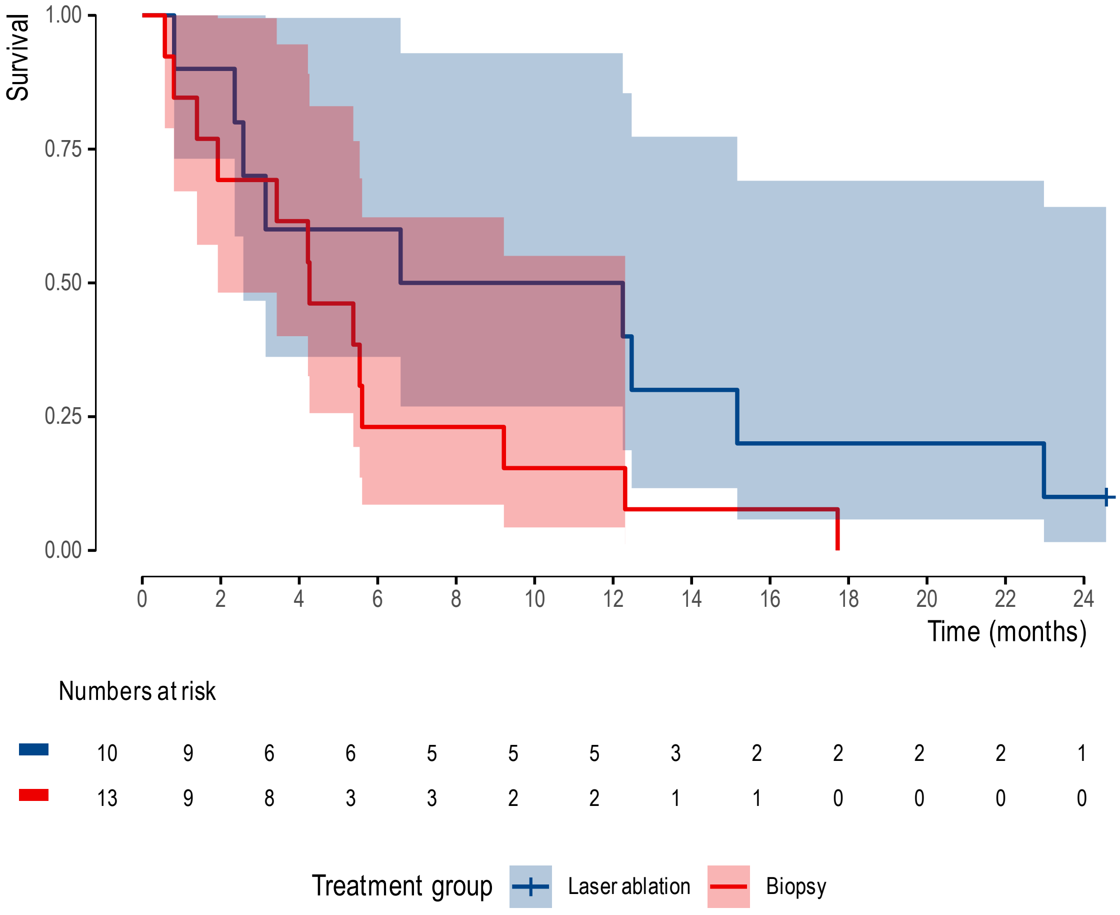

Supplementary item 3: Individual patient data

Patient number	Randomisation group	Age (years)	Baseline tumor volume (cc)	Tumor location	Baseline KPS	Length of hospital stay (days)	Adjuvant therapy	Survival (months)	Quality of Life at 5 months
110001	Intervention	61	8.2	Insula (R)	90	2	None	0.81	NA
110002	Control	77	14	Parietal (L)	80	2	Temozolomide alone	5.54	62.05
110003	Control	71	3.2	Frontal/CC (bilat)	90	2	None	5.60	69.06
110004	Intervention	56	5.73	Thalamus (L)	90	2	None	2.36	NA
110005	Control	70	13.8	Parietal (R)	90	2	Stupp Protocol	12.31	82.69
110006	Intervention	73	3.94	Temporal (R)	90	5	Elderly Schedule	12.47	74.83
110007	Intervention	69	17.8	Temporal (R)	90	3	Stupp Protocol	24.56 *	81.28
110008	Control	71	38	CC (central)	70	2	None	1.39	NA
120002	Intervention	64	31	Frontal (bilat)	80	2	Radiotherapy alone	9.05	98.72
120003	Control	50	4	BG/Thalamus/Brainstem (R)	90	4	None	1.93	NA
130001	Control	76	10.7	Temporal (L)	90	3	Elderly Schedule	4.26	NA
130003	Intervention	42	0.84	Occipital (L)	90	3	Stupp Protocol	22.98	87.78
130004	Control	52	27.1	CC (central)	80	18	None	0.81	NA

130005	Control	67	19.5	BG (R)	80	2	None	9.22	71.07
130006	Intervention	63	3	Frontal (L)	90	2	Stupp Protocol	15.16	86.24
130007	Intervention	75	1.90	Frontal (R)	100	4	Elderly Schedule	6.58	62.56
130008	Intervention	75	17	BG (L)	80	2	None	0.88	NA
140001	Control	68	7.37	BG (L)	80	2	Elderly Schedule	3.43	NA
140002	Intervention	73	7	CC (central)	70	3	None	3.14	NA
140003	Intervention	75	2.07	Parietal (L)	80	5	None	2.58	NA
140004	Control	71	5.39	BG (L)	70	2	None	4.22	NA
140005	Control	78	20.8	Temporal (L)	70	2	None	0.57	NA
150001	Control	59	18.2	Thalamus (L)	90	2	Temozolomide alone	5.38	ND
160001	Control	63	6.23	BG (L)	100	2	Stupp Protocol	17.72	91.03
160002	Intervention	60	3.42	Thalamus (R)	70	4	Stupp Protocol	12.25	76.75
170003	Control	51	20.2	BG (R)	90	2	Stupp Protocol	16.74 *	86.54

No patient was admitted to the Intensive Care Unit. Quality of Life at 5 months after randomization is presented as the EORTC-QLQ-C30 summary score. NA = not applicable; ND = no data. An asterisk (*) indicates that the patient was alive at the time of analysi
